# Comparative Evaluation of CNN and Transformer-Based Instance Segmentation Architectures for Whitefly Detection in Citrus Crops

**DOI:** 10.3390/plants15182874

**Published:** 2026-09-19

**Authors:** Luis Manzano-Soto, Christian Fernández-Campusano, Humberto Verdejo-Fredes, Cristhian Becker, Raúl Carrasco

**Affiliations:** 1 Department of Electrical Engineering, Faculty of Engineering, University of Santiago de Chile (USACH), Santiago 9170124, Chile; luis.manzano@usach.cl (L.M.-S.); humberto.verdejo@usach.cl (H.V.-F.); cristhian.becker@usach.cl (C.B.); 2 Departamento de Contabilidad y Gestión Financiera, Facultad de Administración y Economía, Universidad Tecnológica Metropolitana, Santiago 7500998, Chile; rcarrascoa@utem.cl

**Keywords:** smart farm, precision agriculture, pest detection, computer vision, *Aleurothrixus floccosus*

## Abstract

This study focuses on the detection of pests in citrus fruits using computer vision (CV) techniques and image detection technologies based on convolutional neural networks (CNNs). Early detection enables pest control, reduces the use of pesticides, and generates alerts for the inspection of surrounding areas. One of the models used is You Only Look Once (YOLO), which is based on a single-stage CNN. This model is fast, resource-efficient, and has low latency, enabling it to run on a personal computer. Another type of neural network is based on the vision transformer, a paradigm that requires more hardware resources than YOLO but offers greater precision. Therefore, a comparison is carried out between CNN models to evaluate their precision and demonstrate that YOLO is an excellent option compared to vision transformer-based models, which are more resource-intensive. Through the capture of images in the field, it will be shown that the difference in precision compared to other models that are more demanding in terms of hardware is minimal. The study also reveals that all the models assessed have difficulties in detecting small objects, suggesting a need to improve this through software techniques or hardware enhancements, such as the optics of drone lenses. A wide-angle lens could be used for preliminary detection, and another with a higher zoom for confirmation and the detection of small insects, thereby facilitating its adoption where computational resources are limited.

## 1. Introduction

*Aleurothrixus floccosus*, commonly known as the woolly whitefly, is a significant pest affecting citrus crops in various countries, including Chile [1], Spain [2], India [3], and South Africa [4]. Their nymphs are oval and flattened in shape and are covered by a white secretion that acts as a protective barrier, whilst the adults have a yellowish body covered by a powdery white wax that gives them a woolly, spongy appearance. These microscopic insects generally inhabit the undersides of leaves and feed on sap, causing weakening, leaf drop, stunted growth, and an increased likelihood of saprophytic fungal proliferation [5]. As a result of the above, this poses a problem in infested plantations as it often requires the excessive use of insecticides, which increases economic costs and raises environmental concerns [6].

Nowadays, the use of computer vision combined with unmanned aerial vehicles (UAVs) such as drones has emerged as a viable alternative in precision agriculture [7,8], and several recent studies have applied deep learning to the recognition of pests, weeds, and diseases in various crops, including soya [9], rice [10], maize [11], potatoes [11], tobacco [12], and peppers [13]. Although humans with expertise in their crops can easily identify the pest through visual inspection, this approach is labour-intensive and impractical for large plantations or in areas that are difficult to access. For this reason, this study proposes a comparison of four computer vision models: two from the YOLO family, which offer low latency and lower hardware requirements, and two models with high hardware demands, such as the Mask Region-based Convolutional Neural Network (Mask R-CNN) and RF-DETR-SEG M, which have longer processing times and consequently higher latency. The aim is to test whether there is a significant gap in precision and recall between these models. If the gap between them is not very large, it is entirely plausible to use the YOLO models for real-time detection and the more hardware-intensive models for pre-annotation tasks. To this end, metrics such as precision and recall, latency, and hardware consumption of the models will be taken into account. For citrus crops in particular, a previous study [14] demonstrated the feasibility of using a YOLO v10s detector running on a personal computer to process video streamed by a low-cost UAV, reporting an average precision of 76% on a test set of static images. Bounding box-based object detection methods [15] are sufficient when the aim is to indicate the presence of the pest within a region of interest. However, several subsequent tasks of practical interest require pixel-level localization rather than rectangular boxes [16]. Counting individuals in dense clusters, estimating the proportion of leaf area covered by infestation, or providing high-quality pre-annotations to speed up the construction of larger datasets are tasks that benefit from the use of instance segmentation masks. This work therefore focuses on segmentation models.

Three families of architectures dominate the current literature on instance segmentation. Single-stage models, exemplified by the YOLO family [17,18,19,20,21], predict object boundaries, classes, and masks in a single forward pass, offering high inference speed at a moderate computational cost. Two-stage models, exemplified by Mask R-CNN [22] and implemented in mature frameworks such as Detectron2 [23], first generate region proposals and then refine the bounding boxes and masks within each proposal, typically at the cost of additional latency in exchange for higher localization quality. More recently, end-to-end transformer-based models [24], exemplified by the DETR family and its real-time variants such as RF-DETR [25,26], eliminate anchor heuristics and non-maximum suppression (NMS) and predict bounding boxes, classes, and masks directly from a set of queries learned on a transformer backbone (typically DINOv2), offering a new trade-off between precision and latency.

### Contributions

The contribution of this work is twofold. Firstly, we present a controlled comparison of four modern instance segmentation architectures applied to the same whitefly dataset under identical training and evaluation conditions (YOLO11s-seg, YOLO26s-seg, Mask R-CNN, and RF-DETR-Seg (Medium)), thereby covering the three dominant instance segmentation paradigms: single-stage CNN-based, two-stage CNN-based, and vision transformer-based. Secondly, we report the standard common objects in context (COCO) metrics [27], including a stratified breakdown by size of the detected instances, defined as average precision small (APs), average precision medium (APm), and average precision large (APl). This highlights the practical limitations of detecting small objects in this task, and motivates future work on resolution, tiling strategies, dataset expansion, and the necessity of a dedicated optics to focus on small objects.

The use of these computer vision models on smart farms enables the automation of detection, replacing the manual work of field staff who must identify pests with the naked eye; while a worker may perform well at the start of their shift, accumulated fatigue after long hours of work can reduce detection effectiveness. Moreover, it has been demonstrated that small CV models can work well in real-time applications because of their lower consumption of resources and good latency.

Further motivation for the use of CV models include the fact that they prevent massive sap loss, protecting the citrus tree’s vigor and avoiding premature defoliation, while enabling successful biological control by allowing natural enemies to act before the pest forms its waxy barrier. It reduces operating costs and environmental impact by allowing preventive, localized treatments instead of mass spraying.

There is no instance segmentation study for *Aleurothrixus floccosus* that includes a controlled comparison of the three CNN paradigms (single-stage, two-stage, vision transformer) using the same evaluation metric on a pest dataset; furthermore, this study reports counting error alongside precision and latency.

## 2. Related Work

### 2.1. Pest Detection in Agricultural Imagery

The application of computer vision to the detection of agricultural pests has grown rapidly in recent years. Early work was based on classical feature extraction and shallow classifiers, but the field shifted decisively towards deep learning once large labeled datasets became available. Recent surveys [7,18] highlight the predominance of convolutional architectures and, more specifically, the YOLO family as a flexible baseline for real-time deployment. Works such as [10,11] apply variants of YOLO to diseases affecting rice leaves, maize, and potatoes, respectively, all of which share our problem—the challenge of dealing with small, partially occluded targets against a textured background.

For citrus fruits in particular, the use of static images and UAV-mounted cameras has been explored in [14], where a YOLO v10s detector was trained on 500 augmented images and evaluated both offline and on a real-time stream. Whilst that work establishes the feasibility of the baseline, it operates in a detection scenario (bounding boxes) and does not report segmentation metrics. The present study complements that line of work by focusing on pixel-level masks.

Beyond the cited works, it can be observed that a large number of recent studies are associated with the YOLO family, where there is a tendency to use it for the detection of agricultural pests. There have been multiple variants adapted to the agricultural world published in the last two years, which are proposals of different architectures seeking to solve distinct problems. In the context of small and densely distributed insects, Zhu et al. [28] extended YOLOv8s with a tiny-object detection layer, an asymptotic feature pyramid network, and a hybrid attention transformer module, reaching a medium average precision (mAP) of 94.2% on stored-grain insects. An analogous concern for small targets motivated the low-resolution detector proposed by Wang et al. [29], which integrated a convolutional block attention module in YOLOv8 and reported an mAP@50 of 93.8% against several YOLO baselines (Retina Net and Faster R-CNN). In cotton, Jiang et al. [30] incorporated their dynamic snake convolution model in YOLOv8n to capture the elongated body structure of pests, reaching an mAP@50 of 97.5%. Deployment-oriented variants, such as the Ghost-YOLO-Shuffle Attention detector embedded in the artificial intelligence IoT framework of Li et al. [31], illustrate the strong emphasis of the current literature on efficient and real-time pipelines. Systematic head-to-head evaluations between architectural families, however, remain relatively scarce [32]: offers one of the few systematic comparisons of one-stage (YOLOv8) and two-stage (Mask R-CNN) segmentation in orchards, reporting a clear advantage of YOLOv8 both in precision (up to 0.93) and in inference latency (7.8–10.9 ms vs. 12.8–15.6 ms), while Zhang et al. [33] contrasts an improved variant of YOLOv11 (RDL-YOLO) against RT-DETR, D-FINE, and STN-YOLO on cotton leaf pests. With regard to pest damage in citrus specifically, Ünal and Eceoğlu [34] recently proposed a lightweight YOLO12n-Seg for the simultaneous assessment of fruit ripeness and red scale (*Aonidiella aurantii*) damage, reaching an mAP@0.5 of 0.980 on the pest class. Finally, works that already go beyond bounding boxes towards pixel-level segmentation remain scarce: Şahin et al. [35] evaluated YOLOv8 on eleven tomato pest taxa under commercial field conditions and reported detection and segmentation metrics above 97% in both modalities, being one of the few field-scale benchmarks that jointly quantify both tasks. Taken together, these works confirm the maturity of YOLO-based pipelines for pest recognition, but also expose two consistent limitations: most benchmarks are anchored on tomato, cotton, rice, or stored-grain species, and the pixel-level segmentation of small foliar pests on citrus has not been widely addressed.

### 2.2. Single-Stage, Two-Stage, and Transformer-Based Instance Segmentation

In the broader field of computer vision, single-stage methods, two-stage methods and, more recently, those based on end-to-end transformers have followed parallel trajectories. Mask R-CNN [22] extended Faster R-CNN with a masking branch, and remains a strong baseline thanks to its modularity and the maturity of the open-source code supporting it. The YOLO family has gradually incorporated segmentation capabilities and, in versions 11 and the recently released 26, offers segmentation variants that are competitive in terms of precision whilst maintaining fast inference. A comprehensive review of YOLO’s evolution across its versions is provided in [18]. In parallel, the DETR family [24] introduced an end-to-end paradigm based on transformers that eliminates anchors and non-maximum suppression, predicting bounding boxes, classes, and masks directly from a set of learned queries. Its real-time variants, such as RF-DETR [26], combine a self-supervised pre-trained backbone (DINOv2 [36]) with a deformable attention decoder, achieving competitive precision with latency comparable to that of single-stage models. To the best of our knowledge, no previous work has compared these four specific models on a citrus whitefly dataset.

Recent advances on the two-stage side have focused on improving the backbone and activation of Mask R-CNN to better handle fine-grained plant structures: El Akrouchi et al. [37] coupled Mask R-CNN with an EfficientNet-B7 backbone and Mish activation for the instance segmentation of quinoa panicles, providing a systematic backbone benchmark that reinforces the value of the two-stage paradigm for structured plant targets. On the transformer side, Wang et al. [38] introduced PD-TR—an end-to-end plant disease detector built on a transformer structure and enhanced with BatchFormerV2, the LAMB optimizer, and a complete intersection over union loss—and Hong et al. [39] proposed Light-HortiNet, a lightweight Mobile-Transformer backbone equipped with cross-scale attention and a small-object enhancement branch that runs at more than 20 frames per second (FPS) on Jetson Nano, outperforming lightweight competitors under greenhouse conditions. These results, together with the improvements reported for RT-DETR variants on cotton leaves (see Zhang et al. [33]), illustrate that transformer-based detectors are steadily closing the gap with real-time one-stage models. Nevertheless, the segmentation branch of the DETR family remains much less studied in agricultural imagery than its detection counterpart and, to the best of our knowledge, no published work has evaluated an RF-DETR segmentation head on any citrus pest dataset.

### 2.3. Research Gap and Novelty

The literature summarized above supports three specific gaps that motivate the present study. First, although YOLO variants dominate pest detection benchmarks, the vast majority of the studies are oriented to fruit ripeness or spots on the fruit and not to foliar pests, whose targets are one or two orders of magnitude smaller and share colour and texture with the leaf surface: the only closely related exception, [34], addresses red scale on the fruit and not whitefly on the leaf and does so with a single architecture. Second, no systematic comparisons have been reported that evaluate, on the same dataset and protocol, a two-stage baseline (Mask R-CNN), the most recent one-stage YOLO segmentation variants (YOLO11-seg, YOLO26-seg), and a transformer-based segmenter (RF-DETR-Seg) for whitefly. The existing comparative benchmarks either omit the transformer family [32] or restrict themselves to bounding-box detection [33]. Third, the published RF-DETR results in agriculture have so far been limited to detection tasks, leaving the pixel-level performance of its segmentation head uncharacterized in this domain. The present work addresses these three gaps simultaneously: we evaluate YOLO11s-seg, YOLO26s-seg, Mask R-CNN, and RF-DETR-Seg M on a new pixel-level annotated dataset of *Aleurothrixus floccosus* on citrus leaves. In doing so, we extend the line of work on citrus pest detection exemplified by Ünal and Eceoğlu [34] towards a foliar pest and instance segmentation, complement the YOLO vs. Mask R-CNN comparison of Sapkota et al. [32] with two additional architectural families (YOLO26-seg and RF-DETR-Seg), and contribute the characterization of the RF-DETR segmentation branch on a citrus pest dataset.

## 3. Materials and Methods

**Figure 1:**
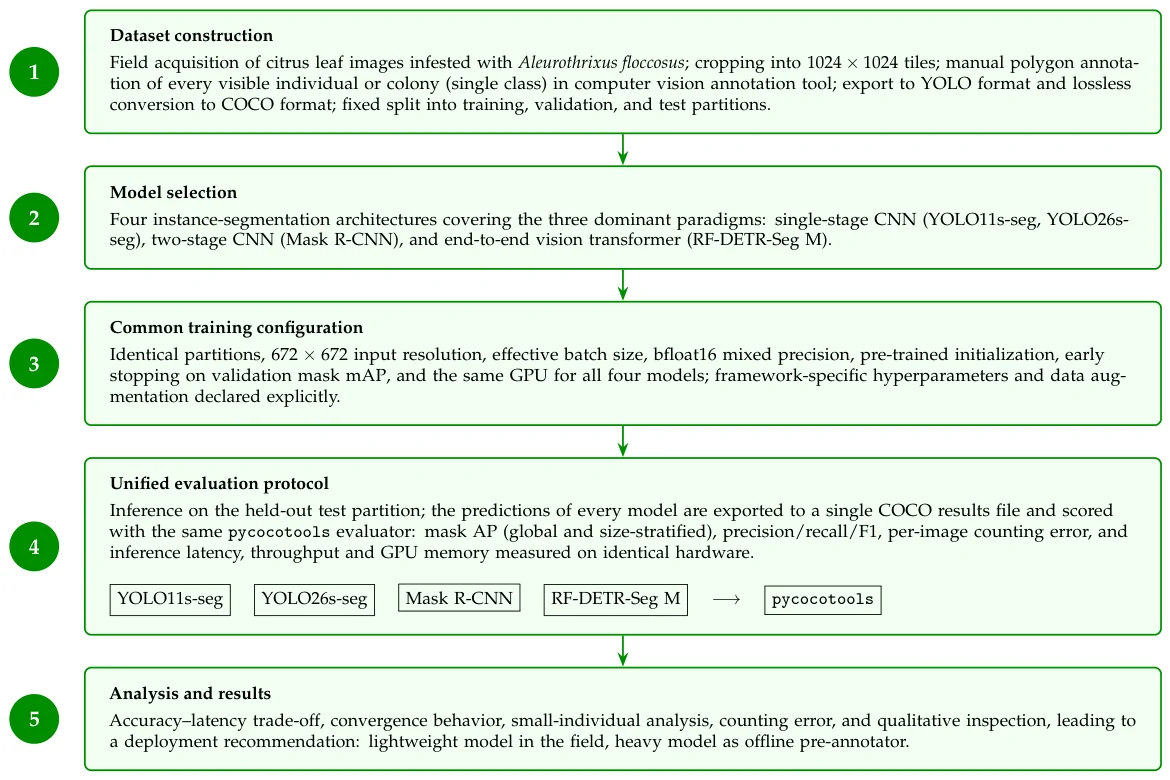
Flowchart of the computer vision pipeline for the detection of Aleurothrixus floccosus.

Figure 1 shows the workflow from the acquisition of field images to the implementation recommendation. Stage 4 highlights that the predictions from the four models are assessed by a single evaluator, so that any differences between the models cannot be attributed to the evaluation procedure.

### 3.1. Dataset

The dataset used in this study consists of 633 high-resolution images of citrus leaves naturally infested with *Aleurothrixus floccosus*, acquired in the field by the authors. Each image was processed as a 1024×1024 tile and manually annotated with polygonal masks delineating each visible individual or cluster of the pest using a single labeled class, whitebug. The annotations were carried out using the cvat tool, which enables polygon-based segmentation, and were exported in the YOLO segmentation format (instance-normalized polygon coordinates); these same polygons were then transformed into the COCO format to ensure compatibility with the Mask R-CNN and RF-DETR-Seg evaluation pipelines.

The 633 annotated images were divided into training, validation, and test subsets. The test set is the most important, as the model never sees it and it allows us to check how well our model generalizes once training is complete. These evaluations are analyzed in Section 4, where the test set contains 61 images and 495 annotated instances of *Aleurothrixus floccosus*; no images from the test set were used at any point during training or validation. The training and validation sets contain 481 and 91 images, respectively, making a total of 633 images in the dataset.

The images were taken at midday on a sunny, cloudless day and over a period of approximately two hours. Images were captured from various angles, including those showing the leaf with greater exposure to the sun and others with more shade, taken beneath the tree to represent low-light conditions. Photographs were taken not only of the leaves, but also of the tree trunks themselves and the sky, so that the model could learn to correctly distinguish what it should classify and what it should not.

Table 1 shows the structure of the dataset; of the total 633 images, 481 are training images, 91 are validation images, and 61 are test images.

Table 2 shows the number of images and their proportions. These were used for training purposes and were divided into three sets: training, validation, and test.

### 3.2. Models Compared

Instance segmentation architectures were trained and evaluated under equivalent conditions (see Table 3).

### 3.3. Training Configuration

The four models were trained under equivalent conditions in order to isolate the effect of the architecture from that of the training methodology.

#### 3.3.1. Hyperparameters Common to All Four Models

All models were trained with a standardized input resolution of 672×672 pixels (due to the stride constraint of the YOLO models and the patch constraint of RF-DETR-Seg), with an effective batch size of 16, using mixed-precision bfloat16, and on the same NVIDIA A100-SXM4 Graphics Processing Unit (GPU) with 40 GB of video random access memory (VRAM), provided by Google Colab. The four training runs were performed on the same dataset and against the same validation partition.

#### 3.3.2. YOLO11s-Seg and YOLO26s-Seg

Both models were trained with virtually identical hyperparameters using the Ultralytics framework: a physical batch size of 16 (equivalent to the target effective batch size), an initial learning rate of ${\mathrm{lr}}_{0}=0.01$ with a linear schedule to ${\mathrm{lr}}_{f}=0.01$, automatic mixed precision (amp = True), and a conservative augmentation scheme for small objects (probability of mosaic reduced to 0.5, mosaic disabled in the last 10 epochs, copy-paste disabled, maximum rotation of 5°, horizontal and vertical flipping with a probability of 0.5 and subtle variations in HSV). The optimizer was set to auto, which selects SGD for YOLO11s-seg and MuSGD for YOLO26s-seg. Early-stopping was set with a patience of 20 epochs based on the validation partition’s mask mAP. The maximum number of epochs was set to 120 for YOLO11s-seg and 130 for YOLO26s-seg.

#### 3.3.3. Mask R-CNN

Mask R-CNN was implemented in Detectron2 [40] using the mask_rcnn_R_50_FPN_3x configuration, and initialized with the model and its pre-trained weights from COCO. To match the effective batch size of 16 used by the other models, IMS_PER_BATCH = 16 was set, which, with a training batch size of 625 images, equates to 39 iterations per epoch. The target of 100 epochs resulted in MAX_ITER=3900 iterations, with a base learning rate of 0.01 and learning rate reduction steps at 70% and 90% of the total training distance. The input resolution was set to 672×672 for both training and inference (INPUT.MIN_SIZE_TRAIN, MIN_SIZE_TEST and the corresponding MAX_SIZE values, both set to 672). Automatic mixed precision (AMP) was enabled (SOLVER.AMP.ENABLED = True) in bfloat16. The only non-standard component in Detectron2 was an additional early-stopping hook with a patience of 20 epochs on the validation segm/AP, which triggers stopping and saves the best checkpoint (model_best.pth) whenever the metric improves; this allows the optimal model to be recovered even when training is interrupted before MAX_ITER.

#### 3.3.4. RF-DETR-Seg (Medium)

RF-DETR-Seg (Medium) was trained using the official Roboflow framework for a maximum of 100 epochs with a learning rate of $1\times 10^{-4}$. To achieve the target effective batch size of 16 without exceeding the available VRAM, a physical batch size of two with eight-step gradient accumulation was used (2×8=16). The framework uses AMP in bfloat16 by default. Early stopping was configured with a patience of 20 epochs and a minimum delta of 0.001 on the validation mAP calculated from the exponential moving average (EMA) weights, which is the criterion selected by the framework itself by default. Under this configuration, training converged early and triggered early stopping around epoch 35, before reaching the limit of 100 epochs. The evaluation of RF-DETR-Seg was carried out on the checkpoint checkpoint_best_total.pth, which the framework automatically selects as the best among the regular weights and the EMA weights based on the validation mAP; in this run, the EMA weights marginally outperformed the regular weights (validation mAP of 0.5111 vs. 0.5052), and were therefore selected.

The same set of images was used for all frameworks; pre-processing consisted solely of cropping the original images to give all the images used a resolution of 1024 × 1024 pixels—the hyperparameters are listed in Table 4, which shows the evaluation protocol. This was carried out using the PyCocoTools tool, a Python (version 3.13.15) library that helps to evaluate models in a standardized manner. The first aspect is data augmentation: each framework implements its own pipeline, and not all of them employ the same operations. In YOLO, a conservative scheme was used for small objects; Detectron2 applies only horizontal flipping by default; and RF-DETR-Seg applies multi-scale training with square rescaling to 672, with horizontal flipping disabled by the framework itself. This asymmetry may favour the YOLO models, which received the richest image augmentation but still underperformed compared to RF-DETR-Seg; this reinforces the conclusion that the more hardware-demanding architectures detect slightly more than YOLO, as demonstrated in this paper. Nonetheless, the most powerful architecture was not as extensively augmented as those in YOLO.

#### 3.3.5. Equipment and Training Times

The four experiments were run on the same Google Colab Pro instance with a 40 GB NVIDIA A100-SXM4 GPU. The choice of the A100 over more economical alternatives (T4, L4) was driven by the VRAM requirements of RF-DETR-Seg at 672×672 with a physical batch size of 2, which peaked at ∼26 GB during training—beyond the capacity of a 24 GB L4. Table 5 summarizes the total times and peak resource usage recorded for each model through periodic sampling.

The running times show a marked asymmetry: the three CNN-based models converged in less than half an hour, whilst RF-DETR-Seg took approximately two and a half hours. This difference is mainly due to the higher computational cost per iteration of the transformer decoder and the smaller physical batch size (2 vs. 16) required to comply with the VRAM constraint, which results in a greater number of optimizer steps and lower average GPU utilization.

### 3.4. Evaluation Protocol

The four models were evaluated on the reserved test set (61 images, 495 instances) using the standard COCO instance segmentation metrics [27]: AP@0.5 (mAP50), AP@0.5:0.95 (mAP), AP@0.75, and the size-stratified variants APs, APm, and APl. Detections were not post-processed beyond the default NMS of each framework; in the case of RF-DETR-Seg, the model is natively NMS-free, so predictions were obtained directly from the set of learned queries without an additional suppression stage.

To ensure comparability, the metrics for the four models were calculated using the same evaluator. To this end, the predictions from each model were exported to COCO format (file predictions_test.json) and processed using the official pycocotools.COCOeval interface, utilizing the same ground truth file (instances_test.json). This eliminates any bias that might be introduced by heterogeneous evaluators across different frameworks.

#### Counting Error Metrics

In addition to the COCO metrics, the instance counting error per image is evaluated by treating the count as a regression problem, in line with standard practice in the literature on visual counting [41]. Let $y_{i}$ be the number of instances annotated in image *i* and ${\hat{y}}_{i}$ the number of detections produced by the model after filtering out predictions with a confidence score below τ=0.25; the same threshold is applied to all four models. Three complementary error metrics are computed on the N=61 images in the test partition:

The Mean Absolute Error (MAE) measures the average magnitude of the counting error per image in the same units as the observed variable (instances). It is robust to extreme values and is the primary metric in most visual counting studies [41]:

(1) $$MAE=\frac{1}{N}\sum_{i=1}^{N}\left|y_{i}-{\hat{y}}_{i}\right|.$$

The Root Mean Square Error (RMSE) also measures error in terms of instances, but penalizes large deviations quadratically; it is therefore more sensitive to images with outlier errors and complements the MAE by highlighting the variance of the error [41]:

(2) $$RMSE=\sqrt{\frac{1}{N}\sum_{i=1}^{N}{\left(y_{i}-{\hat{y}}_{i}\right)}^{2}}.$$

The Mean Absolute Percentage Error (MAPE) expresses the counting error relative to the true value, providing a scale-independent measure that facilitates interpretation when the density of instances varies significantly between images [42]:

(3) $$MAPE=\frac{100}{N}\sum_{i=1}^{N}\frac{\left|y_{i}-{\hat{y}}_{i}\right|}{y_{i}}\;\left[\%\right].$$

The MAPE is well-defined in our case, as $y_{i}\geq 1$ for every test image.

These three metrics capture a failure mode that is not penalized by the COCO metrics: if the model splits a dense cluster labeled as a single instance into multiple separate detections, the mask average precision (AP) may remain competitive whilst the MAE, RMSE, and MAPE increase simultaneously. The contrast between the MAE and RMSE also provides insight into the homogeneity of the error: similar values indicate stable errors across images, whilst RMSE≫MAE indicates the presence of outlier errors in a few images.

## 4. Results

### 4.1. Quantitative Comparison

Table 6 summarizes the COCO detection (bounding box) and segmentation (mask) metrics obtained by each model on the test set (61 images, 495 instances), all evaluated using the same pipeline based on pycocotools. Precision and recall are reported at the operating point of maximum F1 score at intersection over union (IoU) = 0.5.

The four models fall within a narrow range: the AP for masks ranges from 0.538 (YOLO11s-seg) to 0.593 (RF-DETR-Seg M), a difference of 5.5 points, which narrows to 5.1 points in the AP@0.5 test. RF-DETR-Seg M performs best across all global metrics and offers the best balance between precision and recall (F1 = 0.822). Among the convolutional models, Mask R-CNN achieves the highest AP (0.560), closely followed by YOLO26s-seg (0.540) and YOLO11s-seg (0.538). At the maximum F1 score, YOLO11s-seg is the most accurate (0.882) but has the lowest recall (0.720); RF-DETR-Seg M has the highest recall (0.800) with competitive accuracy (0.845). This determines the choice of model: if the application penalizes false alarms, YOLO11s-seg is preferable; if it penalizes undetected individuals, RF-DETR-Seg M is preferable.

In the stratification by size shown in Table 6, the AP drops by more than 25 points between medium-sized instances APm (0.626–0.679) and small instances APs (0.345–0.410) across all four models; this shows that all models struggle with small instances. The overall AP can be approximated as the average of the three instance-size strata, weighted by the number of instances; thus, the 31% of small instances drag down the results of all models equally. This indicates that the main barrier to detection is the apparent size of isolated individuals at 672 × 672 pixels, not the architecture itself. As these isolated individuals are precisely those that characterize an incipient outbreak, Table 7 examines the small size class in detail. For small individuals, a low APs may be due to three causes: that the model fails to detect the small individuals; that it detects them but with low confidence, causing them to be confused with false positives; or that it detects them but fails to delineate their contours correctly. The data allow us to distinguish between these causes.

Small objects are indeed detected. If all detections with a low confidence threshold—for example, ≥0.05—are accepted, between 79% and 91% of the 154 small instances are assigned a mask with IoU ≥ 0.5; RF-DETR-Seg M detects 140 of the 154. The omission of objects is not the main cause.

The main reason is the confidence that the model assigns to these detections. Correct masks on small instances receive low scores, on par with false positives, whilst masks on medium-sized clusters receive high scores. This is why there is no confidence threshold that distinguishes correct predictions from errors in the small-instance stratum: with a high threshold, instances are missed, and at the point of the maximum F1 score, the recall for small instances is only 0.56–0.66 compared to 0.85–0.86 for medium-sized ones; with a low threshold, false positives are included. The AP, which is calculated across all thresholds, penalizes precisely this lack of separation. This is where RF-DETR-Seg M gains its advantage: its APs@0.5 score is 9–10 points higher than that of the other three models (0.739 vs. 0.640–0.649); in other words, it assigns a higher confidence score to small instances that are genuinely small, which can be attributed to the context provided by the global attention across the entire image.

The accuracy of the pest contour detection is low across all four models. The average IoU for small masks that are correct is 0.76 in all cases, compared with 0.84 for medium masks; when requiring IoU ≥ 0.75, the APs@0.75 falls to 0.400–0.412 for all four models, with no advantage for any of them. With masks comprising just a few pixels, each pixel of error at the edge disproportionately reduces the IoU; this limitation stems from the image resolution, not the architecture. The advantage of RF-DETR-Seg M over small instances therefore lies in detection confidence, not mask quality. This could be resolved through hardware solutions or higher-resolution images.

In terms of point-wise precision and recall (operating at F1-max), YOLO11s-seg achieves the highest precision for both masks (0.882) and bounding boxes (0.883), but at the cost of a significantly lower recall (0.720). RF-DETR-Seg, at the opposite end of the spectrum, achieves the highest recall (0.800) with a competitive precision (0.845), offering the best overall precision–recall trade-off. This trade-off determines operational use: if the application heavily penalizes false alarms, the YOLO models are preferable; if it penalizes false negatives, RF-DETR-Seg is the safer option.

The breakdown by size according to the COCO convention also confirms that the detection of small individuals remains the greatest challenge for all four architectures. The mask APs obtained are 0.345 (YOLO11s-seg), 0.346 (YOLO26s-seg), 0.372 (Mask R-CNN), and 0.410 (RF-DETR-Seg), compared with values ranging from 0.625 to 0.679 for medium-sized objects and from 0.587 to 0.648 for large objects. The drop of more than 25 points between APs and APm persists even for the best-performing model evaluated, suggesting that the dominant bottleneck in this task is the apparent size of individual whiteflies within the tiles, rather than the choice of architecture.

**Figure 2:**
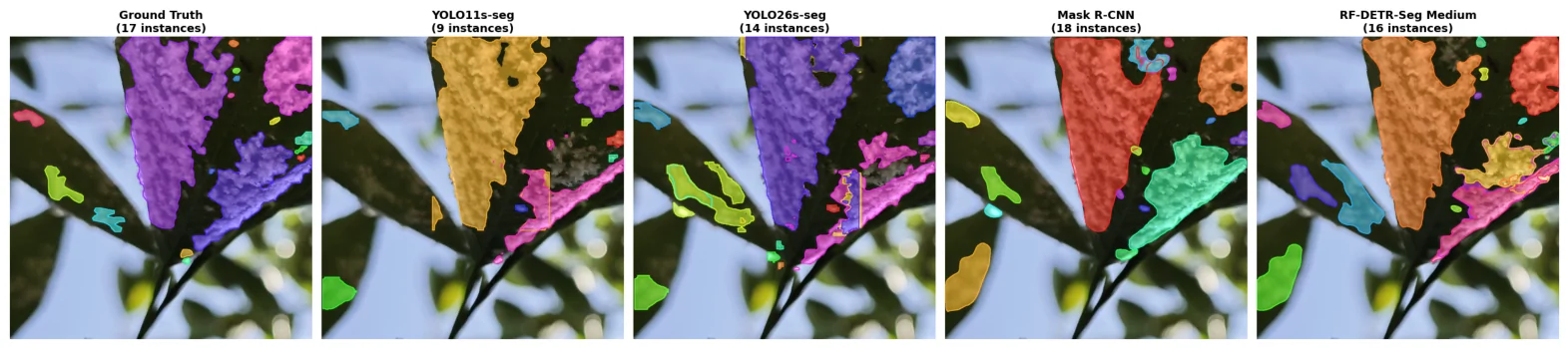
Visual comparison between the ground truth and the predictions generated by the four models evaluated (YOLO11s-seg, YOLO26s-seg, Mask R-CNN, and RF-DETR-Seg Medium) for the same test image.

Figure 2 shows, in different colours, how the models are able to detect the segmentation of the pest on the leaf; the models are able to detect the pests, but where they fall short is in counting the number of clusters present. However, this is debatable, as the pests to be detected are quite diffuse. This raises the question regarding where one ends and the other begins. All the models, however, detect the pests correctly when compared to the ground truth.

### 4.2. Convergence Behavior

**Figure 3:**
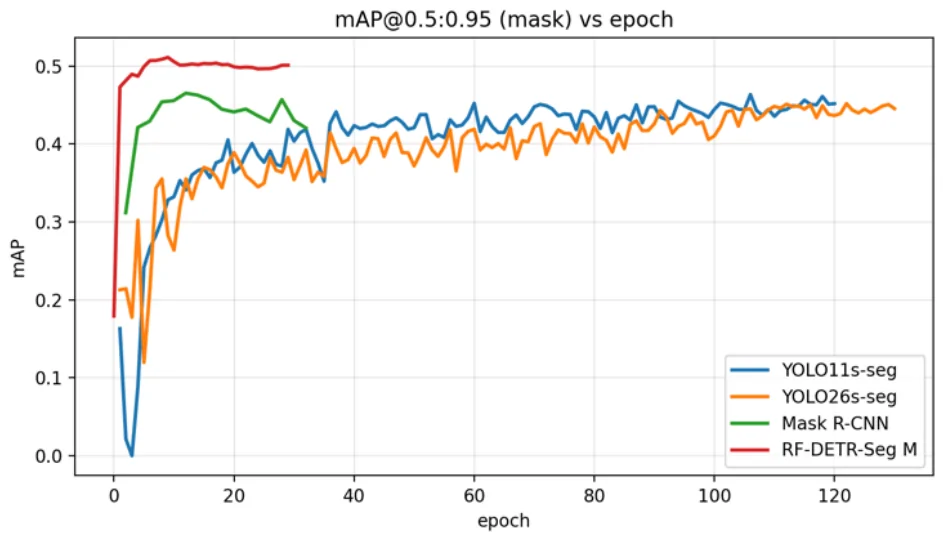
Convergence of the masked mAP@0.5:0.95 on the validation partition throughout training. RF-DETR-Seg and Mask R-CNN converge in fewer than 15 epochs and are then stopped as they fail to show significant improvements in AP; the YOLO models require more than 100 to reach a comparable level.

Figure 3 shows the mask mAP@0.5:0.95 on the validation partition throughout training. RF-DETR-Seg converges first, reaching its best validation mAP (≈0.51) around epoch 9 and triggering early stopping around epoch 29. Mask R-CNN also converges quickly, reaching its best performance (≈0.47) around epoch 12. In contrast, the YOLO models require a significantly longer training process: YOLO11s-seg only reaches its best validation mAP (≈0.46) at epoch 106, and YOLO26s-seg (≈0.45) at epoch 122. In other words, YOLO models require approximately an order of magnitude more epochs to achieve a validation mAP that is practically equivalent to that of Mask R-CNN and only a few points below that of RF-DETR-Seg. This difference is consistent with the nature of the pre-training used by each model (DINOv2 SSL in RF-DETR-Seg, supervised COCO in Mask R-CNN, and Ultralytics’ pre-COCO weights in YOLO).

### 4.3. Inference Efficiency

Table 8 summarizes the inference cost measured under equivalent conditions: the same NVIDIA A100 GPU, the same input resolution of 672 × 672, and the same set of test images.

**Figure 4:**
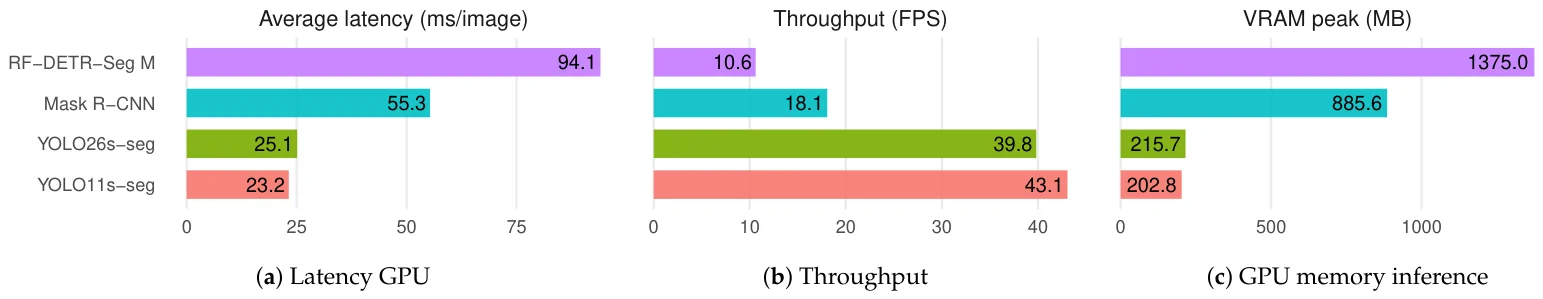
Latency per image, throughput, and peak VRAM usage during inference at 672 × 672 on an NVIDIA A100.

Looking at Figure 4, we can see that the YOLO models have the lowest latency, making them ideal for real-time monitoring. In terms of frames per second, the YOLO models once again deliver the best frame rate, reaffirming their suitability for real-time monitoring; finally, in terms of memory consumption, the best models are once again the YOLO models. This suggests that YOLO is the best option for real-time deployment. However, for pest annotation and reinforcement learning, the heavier RF-DETR-Seg M model can be used. Whilst it is not feasible for use in edge computing, it is suitable for pre-annotation.

The differences in inference cost are much greater than the differences in precision. YOLO models are approximately 2.4× faster than Mask R-CNN and 4× faster than RF-DETR-Seg, and consume between 4.4× and 6.8× less VRAM than these. With peak VRAM usage below 220 MB and a throughput of around 40  FPS on the A100, both YOLO models are the only viable candidates for deployment on low-cost edge devices (for example, a Jetson unit mounted on a drone), whilst Mask R-CNN and RF-DETR-Seg are restricted to processing or annotation on a workstation with a dedicated GPU.

### 4.4. Counting Error and Qualitative Observations

Table 9 summarizes the counting error per image on the test partition. YOLO26s-seg achieves the best results across all three metrics (MAE = 2.46, RMSE = 3.10, MAPE = 55.3%), whilst Mask R-CNN achieves the worst (MAE = 5.18, RMSE = 7.31, MAPE = 158.3%), indicating a strong tendency towards over-detection due to cluster fragmentation; that is, a single cluster is treated as several separate instances. One pattern worth noting is that the ranking between RF-DETR-Seg and YOLO11s-seg depends on the metric: RF-DETR-Seg achieves a better MAPE (76.5% vs. 83.9%) but a worse MAE (3.84 vs. 3.25), suggesting that YOLO11s-seg makes smaller absolute counting errors on average at the cost of a few isolated images where the relative error spikes, whilst RF-DETR-Seg maintains a more consistent relative error, albeit with a slightly higher MAE. Visual inspection of the predictions is consistent with these figures: the three CNN-based models tend to fragment large areas of the infested leaf (dense clusters) into multiple smaller detections; in images with scattered individuals, by contrast, the count is close to the ground truth. Mask R-CNN is the model most affected by this effect and records the worst MAPE (158%), whilst YOLO26s-seg handles it better (MAPE of 55%) thanks to its higher precision. In YOLO masks, a straight-edge artifact is also observed in large masks, corresponding to the clipping of the mask against the bounding box; this artifact does not appear in either Mask R-CNN or RF-DETR-Seg, whose masks are generated at the proposal level or at the full-image level, respectively.

These two patterns give rise to an empirical observation relevant to future work: the current dataset labels both isolated individuals and dense clusters of several overlapping individuals as a single instance, which forces the model to make an arbitrary decision between fragmenting the cluster or predicting a single large mask. A reformulation of the problem with two classes—bug for isolated individuals and cluster for dense clusters—would likely resolve the ambiguity naturally, reduce the counting error, and allow separate metrics of infestation severity to be reported, without requiring a change in architecture.

### 4.5. Trade-Offs and Practical Recommendations

When considering precision, inference cost, and convergence behavior, the four architectures fall into two groups with clearly distinct profiles. RF-DETR-Seg offers the best overall performance (a mask mAP@0.5:0.95 of 0.593 and the highest recall), but at an inference cost 4× higher than YOLO and with peak VRAM usage of around 1.4 GB. The YOLO models offer precision just ∼5 points lower (mask mAP@0.5:0.95 ≈ 0.54) at a fraction of that cost: ∼23 ms per image, ∼200 MB of VRAM, and an order of magnitude fewer parameters. Mask R-CNN occupies a middle ground and does not outperform any of the other three models on any particular metric.

Given that the precision gap between the YOLO and RF-DETR-Seg models is small in absolute terms, whilst the gap in inference cost and memory consumption is much more pronounced, the YOLO models are the recommended choice for operational deployment—particularly for edge and UAV scenarios where latency and VRAM are real constraints—whilst RF-DETR-Seg is reserved as a precision benchmark and as an offline pre-labeler to accelerate dataset growth.

## 5. Discussion

The most suitable model for use in agricultural settings is YOLO, as the inherent physical limitations of the devices—such as, in the case of drones [43,44], their limited payload capacity and battery life—mean that a domestic drone, for example, would be unable to carry a commercial GPU. Therefore, priority should be given to models that can run on lighter hardware with more modest inference capabilities; YOLO is a very low-latency, single-stage model that consumes few VRAM resources—around 200 megabytes, as seen in this study [45]. This model meets the requirements for real-time monitoring in agricultural environments; however, drone flights may take place in enclosed environments where the side passages between trees are 3 to 4 metres wide and surrounded by branches, which is why small devices must be used. These physical constraints—such as unfavorable terrain conditions for flight and payload limitations—are the main factors driving [46] the use of small CV models, such as YOLO, for pest detection.

The detection and management of *Aleurothrixus floccosus*, a pest that causes damage to citrus crops in Peru, has focused on the successful application of modern techniques that combine random sampling and advanced image processing [47]. Sophisticated algorithms have been used to classify images, reduce noise, and smooth edges, resulting in accurate pest detection [48]. Furthermore, Hemipteran’s parasitism by other organisms, such as a hyperparasitoid, has been studied, as this significantly affects the population of *Aleurothrixus floccosus* in different agricultural sectors [49].

In situations where common pests become invasive, as is the case with woolly whiteflies and their complex interactions with other insects, biological control plays a crucial role. Agents of this type, such as Encarsia cubensis in India, have proven effective in combating invasive species [50]. In other parts of the world, such as Italy and Brazil, more comprehensive strategies are being explored, including the management of specific parasitoid agents to reverse the presence and damage caused by *Aleurothrixus floccosus* [51,52].

Morphological and molecular analyses reveal specific details that help to identify and classify the different types of whiteflies found on various crops [53,54]. These studies have direct implications for the implementation of quarantine and control measures to prevent the unauthorized introduction of invasive species, and thus ensure the biosecurity of the global agricultural sector.

A range of innovative solutions has been proposed to tackle the challenge of detecting agricultural pests. MDP-YOLO offers a significant improvement in precision and computational efficiency by reducing the model’s complexity through advanced algorithms, such as lightweight complementary residual, EPConv, Ghost, and WIoUv3 [55]. Furthermore, the YOLO-PEST system has been developed using state-of-the-art techniques, including the use of images captured by UAVs [56], enabling it to outperform other models in terms of precision and efficiency, particularly when compared with neural networks such as DenseNet and FasterNet [57]. RDW-YOLO also makes progress in insect pest detection through key innovations, including RDFBlock for feature extraction, DPDown for multi-scale adaptation, and WWIoU for optimizing bounding box matching and regression [58]. Furthermore, Maize-YOLO stands out for its application in the real-time detection of maize pests using deep CNN technology, representing a substantial improvement in terms of precision and efficiency over other methods [59]. Finally, the PDA-YOLO algorithm has achieved a significant breakthrough in the accurate detection of insect pests in stored grain with high computational efficiency and low cost, proving to be highly effective for urgent tasks in grain storage management [60].

The detection of agricultural pests has advanced significantly thanks to innovative models such as YOLO-DCPG [61], YOLO-CAD based on YOLOv8n [62], and CRRE-YOLO, which have succeeded in advancing pest detection by using lighter architectures to improve precision and reduce computational costs without compromising performance. Furthermore, work such as BEAM-YOLO [63], which combines four innovative modules to increase the separation between the foreground and background, has greatly improved both detection precision and computational efficiency. These advances highlight the relevance of recent studies, such as a portable soya pest recognition system [64] which uses a UAV to capture and pre-process data, thereby optimizing detection with a precision of over 96%. The YOLO-SGInsects [28] and YOLO-PEST [65] models, which use artificial intelligence to detect and count pests, have achieved promising results in terms of precision and efficiency. However, the most notable study has been SP-YOLO [66], a method that significantly improves pest detection in beetroot crops by using a CNN and a transformer to capture global and multi-scale features, achieving an overall precision of 94.9 per cent, which significantly outperforms the original YOLO11 model. These advances not only enhance the ability to detect agricultural pests, but also expand the scope for applying technology in this field, which is vital to global development.

Future challenges in this area include ensuring that models remain resource-efficient, given the nature of edge devices, which have limited resources. In the field, the challenges lie in controlling devices in densely wooded areas, which requires precise drone control, low latency, and the device maintaining its wireless signal for control purposes. Furthermore, varying lighting conditions—such as low light on overcast days—can pose a challenge; it is therefore important to train the models using photographs taken under different conditions so that the chosen model is capable of generalizing well, and can adapt to a variety of situations. The complexity of the background in the captured photos is not a major issue if the model is well trained; however, models suffer from the pareidolia effect of tree trunks, as the trunks have textures similar to the exposed pest infestation.

## 6. Conclusions

This study presented a controlled comparison of four modern instance segmentation architectures applied to the detection of *Aleurothrixus floccosus* on citrus leaves: YOLO11s-seg, YOLO26s-seg, Mask R-CNN, and RF-DETR-Seg (Medium). Under equivalent training conditions (resolution 672 × 672, effective batch size 16, NVIDIA A100 GPU) and evaluation conditions (the same pycocotools pipeline), the four models achieved similar performance: the mask-mAP@0.5:0.95e ranged from 0.538 (YOLO11s-seg) to 0.593 (RF-DETR-Seg M), a gap of around 5 percentage points between the best and worst models. RF-DETR-Seg led across all global metrics, but at the cost of approximately 4× higher inference cost and ∼6.8× higher VRAM consumption than the YOLO models. Analysis stratified by size further confirmed that the dominant challenge of the task is the detection of small individuals rather than the choice of architecture, as the drop in APs relative to APm exceeds 25 points across all four models.

The practical conclusion that can be drawn from this assessment is straightforward: given that the difference in precision between architectures is small in absolute terms, whilst the difference in inference cost and hardware computational cost is by an order of magnitude, the YOLO models (YOLO11s-seg and YOLO26s-seg) are the authors’ recommended choice for field deployment as a solution to the problem of early pest detection, particularly for edge-computing and UAV scenarios where latency and access to VRAM are real constraints. RF-DETR-Seg, however, retains its usefulness as a baseline model and as a high-precision offline pre-labeler to accelerate the growth of the dataset.

This comparative study revealed that YOLO models are an excellent option for deployment on edge devices, based on everything discussed and demonstrated in this paper. The main limitations of this study are (a) the lack of access to a drone on which to install the model and test it in real time, and (b) the lack of an optics laboratory in which to create and test different types of lenses for drones and address the issue highlighted in this study regarding small targets. It is important that, in future, drones used for pest management be equipped with two lenses: one with a wide field of view for early detection, and one with a longer focal length for focusing on and confirming the pest. The practical contribution of this work lies in the fact that, if real-time deployment for detection is required, YOLO should undoubtedly be the choice.

Future work should prioritize, in the following order: (i) reformulating the annotation scheme by introducing two classes—bicho for isolated individuals and cluster for dense clusters—which is expected to reduce both fragmentation ambiguity and counting error; (ii) expanding the dataset in terms of volume and diversity of infestation stages, using RF-DETR-Seg as an assisted pre-annotater to keep the manual annotation and training effort manageable; (iii) reviewing the input resolution and employing tiling during inference to mitigate the problem of detecting small objects, as observed in APs; and (iv) integrating a single-stage YOLO model into a UAV-based pipeline, in line with [14], to evaluate performance at field level under real-world computational and energy constraints.

## Figures and Tables

**Table 1:** Structure of the dataset.

Set	Image	Percentage
Training	481	75.99%
Validation	91	14.38%
Test	61	9.64%
Total	633	100.00%

**Table 2:** Instances by images and statistics.

Split	Image	n Instance	Mean	Median	Q1	Q3	Max
Training	481	4544	9.45	6	2	12	58
Validation	91	828	9.1	5	3	11	62
Test	61	495	8.11	5	3	12	32

**Table 3:** Comparison of segmentation architectures.

Model	Description
YOLO11s-seg	The small variant of the YOLO version 11 segmentation model, released as part of the Ultralytics framework [21]. The model uses a single-stage backbone architecture with a decoupled segmentation head and generates polygons using a prototype-mask formulation.
YOLO26s-seg	The compact variant of the YOLO version 26 segmentation model, released by Ultralytics in early 2026 [20]. Compared to YOLO version 11, this generation introduces the STAL (Soft Target Aware Label) labeling strategy, an improved prototyping module for segmentation, and an end-to-end inference pipeline without NMS. Its default optimizer is MuSGD, a hybrid Muon-SGD optimizer, which the framework automatically selects when configured with optimizer = ‘auto’.
Mask R-CNN	The classic two-stage segmentation architecture [22], implemented in Detectron2 [40]. The configuration used in this study employs a ResNet-50 backbone with a feature pyramid network, a region proposal network, and the mask head, as defined in the original paper.
RF-DETR-Seg (Medium)	The medium variant of the RF-DETR segmentation model, released by Roboflow under the Apache 2.0 licence and built on the DETR (DEtection TRansformer) family [24,25,26]. Unlike the CNN-based architectures described above, this model is an end-to-end transformer: the backbone architecture is a self-supervised pre-trained DINOv2 Vision Transformer, followed by an encoder–decoder with deformable attention blocks and a masking head inspired by MaskDINO. The model directly predicts bounding boxes, classes, and masks from a set of learned queries, eliminating the anchors and NMS typical of classical detectors. This variant divides the image into 12×12 pixel patches and employs two attention windows; this constraint requires the input resolution to be a multiple of 12×2=24 pixels, which is why a standardized resolution of 672 × 672 pixels was chosen for the comparison between the four models, as 672 is both a multiple of 32 (required by the strides of the YOLO models) and a multiple of 24 (required by the RF-DETR-Seg architecture).

**Table 4:** Training configuration of the four models. Top rows are common across frameworks; bottom ones are framework-specific.

	YOLO11s-Seg	YOLO26s-Seg	Mask R-CNN	RF-DETR-Seg M
Common:				
Framework (version)	Ultralytics 8.4.77	Ultralytics 8.4.77	Detectron2 0.6	rfdetr 1.8.1
Backend	PyTorch 2.11.0 + CUDA 12.8			
Train/val/test split	481/91/61 images			
Input resolution	672	672	672	672
Effective batch size	16	16	16	16 (2 × 8 grad. accum.)
Mixed precision	bf16	bf16	bf16	bf16
Initial weights	yolo11s-seg.pt (COCO)	yolo26s-seg.pt (COCO)	R_50_FPN_3x (COCO)	rf-detr-seg-medium.pt
Early stopping	20 ep., val mask mAP	20 ep., val mask mAP	20 ep., val segm/AP	20 ep., δ = 0.001, val mAP
Evaluated checkpoint	best.pt	best.pt	model_best.pth	checkpoint_best_total.pth
GPU	NVIDIA A100 40 GB			
Optimization:				
Optimizer	SGD (auto)	MuSGD (auto)	SGD	AdamW (default)
Initial → Final LR	0.01 → 0.0001 (linear)	0.01 → 0.0001 (linear)	0.01, ×0.1 at 70% and 90%	$1\times 10^{-4}$
Momentum/weight decay	0.937/$5\times 10^{-4}$	0.937/$5\times 10^{-4}$	0.9/$1\times 10^{-4}$	Default
Warm-up	3 epochs	3 epochs	1000 iter	Default
Max epochs	120	130	100 (3000 iter)	100
Stopping epoch	120	130	32	29
Best epoch (val)	106	122	12	9
Seed	0 (deterministic)	0 (deterministic)	Default	Default
Data augmentation:				
Horizontal flip	*p* = 0.5	*p* = 0.5	Yes	Disabled (framework)
Vertical flip	*p* = 0.5	*p* = 0.5	No	No
Rotation/trans./scale	5°/0.1/0.3	5°/0.1/0.3	No	Multi-scale (792) → 672
Mosaic	0.5 (off last 10 ep.)	0.5 (off last 10 ep.)	No	No
HSV (h/s/v)	0.015/0.3/0.3	0.015/0.3/0.3	No	No
Copy-paste/mixup/cutmix	0/0/0	0/0/0	No	No
Architecture-specific:				
Other	close_mosaic = 10	end2end (no NMS)	RoI batch/img = 128	200 queries; group_detr = 13

**Table 5:** Training times and peak resource usage by model. All training runs were carried out on NVIDIA A100-SXM4-40GB on Google Colab Pro.

Model	Time (h:mm:ss)	RAM max. (GB)	GPU util. (% Mean)	VRAM max. (MB)
YOLO11s-seg	0:16:21	8.2	39.2	9155
YOLO26s-seg	0:24:54	7.7	42.2	10,373
Mask R-CNN	0:12:27	4.3	28.7	7177
RF-DETR-Seg M	2:32:17	7.1	12.3	26,609

**Table 6:** Performance on the test partition (61 images, 495 instances), calculated using the same pycocotools evaluator for all four models. Precision, recall and F1 at the peak of the precision–recall curve at IoU = 0.5. AP@0.5 and AP@0.75: AP requiring a single IoU threshold. AP: The average of the ten IoU thresholds between 0.50 and 0.95, representing the strictest criterion. APs, APm, and APl: The same AP calculated only on small (<32^2^ px, n = 154), medium (32^2^–96^2^ px, n = 284), and large (>96^2^ px, n = 57) annotated instances, where n is the number of instances for each size.

	Bounding Box				Mask (Segmentation)				
Model	Precision	Recall	AP@0.5	AP@0.5:0.95	Precision	Recall	AP@0.5	AP@0.5:0.95	Latency
YOLO11s-seg	**0.883**	0.730	0.846	0.605	**0.882**	0.720	0.839	0.538	23.22
YOLO26s-seg	0.815	0.730	0.822	0.599	0.795	0.750	0.824	0.540	25.11
Mask R-CNN	0.846	0.710	0.815	0.535	0.816	0.740	0.830	0.560	55.35
RF-DETR-Seg M	0.845	**0.800**	**0.876**	**0.618**	0.845	**0.800**	**0.875**	**0.593**	94.09

Note: The best value in each column is shown in bold.

**Table 7:** Mask metrics on the 154 small instances (<32^2^ px). Precision_s_, Recall_s_, and F1_s_: precision, recall, and F1 score on small instances at the point of maximum F1 (IoU = 0.5). APs@0.5 and APs@0.75: AP for small instances using a single IoU threshold. APs: AP for small instances averaged over IoU 0.50–0.95, the same range as in Table 6. The last two rows provide the baseline for medium instances (n = 284).

Model	Precision_s_	Recall_s_	F1_s_	APs@0.5	APs@0.75	APs
YOLO11s-seg	0.731	0.56	0.634	0.644	0.404	0.345
YOLO26s-seg	0.684	0.6	0.639	0.64	0.412	0.346
Mask R-CNN	0.701	0.66	0.68	0.649	0.4	0.372
RF-DETR-Seg M	0.771	0.65	0.705	0.739	0.408	0.41
Reference: medium-sized instances						
YOLO11s-seg	0.88	0.85	0.865	0.925	—	0.626
RF-DETR-Seg M	0.882	0.86	0.871	0.942	—	0.679

**Table 8:** Inference cost at 672×672 on an NVIDIA A100. Average latency per image and peak VRAM usage observed during execution.

Model	Lat. (ms)	FPS	VRAM (MB)	Params (M)
YOLO11s-seg	23.2	43.1	203	10.1
YOLO26s-seg	25.1	39.8	216	11.4
Mask R-CNN	55.3	18.1	886	43.9
RF-DETR-Seg M	94.1	10.6	1375	36.1

**Table 9:** Counting error per image on the test partition (n=61, confidence threshold τ=0.25). MAE and RMSE in units of instances/image; MAPE as a percentage. Lower is better. The best value in each column is shown in bold.

Model	MAE	RMSE	MAPE (%)
YOLO11s-seg	3.25	5.61	83.86
YOLO26s-seg	**2.46**	**3.10**	**55.28**
Mask R-CNN	5.18	7.31	158.32
RF-DETR-Seg M	3.84	6.41	76.51

## Data Availability

Full datasets may be available to academic researchers following the completion of appropriate confidentiality agreements.

## Abbreviations

The following abbreviations are used in this manuscript:

AMP	Automatic Mixed Precision
AP	Average Precision
APl	Average Precision Large
APm	Average Precision Medium
APs	Average Precision Small
CNN	Convolutional Neural Network
COCO	Common Objects in Context
CV	Computer Vision
EMA	Exponential Moving Average
FPS	Frames Per Second
GPU	Graphics Processing Unit
IoU	Intersection over Union
MAE	Mean Absolute Error
mAP	Medium Average Precision
MAPE	Mean Absolute Percentage Error
Mask R-CNN	Mask Region-based Convolutional Neural Network
NMS	Non-Maximum Suppression
RMSE	Root Mean Square Error
UAV	Unmanned Aerial Vehicle
VRAM	Video Random Access Memory
YOLO	You Only Look Once
